# Comment on “The Memory of the Heart”, *J. Cardiovasc. Dev. Dis.* 2018, *5*, 55

**DOI:** 10.3390/jcdd5040060

**Published:** 2018-12-19

**Authors:** Robert H. Anderson

**Affiliations:** Institute of Genetic Medicine, Newcastle University, Newcastle upon Tyne NE1 4EP, UK; sejjran@ucl.ac.uk; Tel.: +44-20-8870-4368

**Keywords:** anatomy, development, helical heart, three-dimensional mesh, ventricular mural architecture

## Abstract

In his recent review for the Journal of Cardiovascular Development and Disease, Cirillo offers a concept for “cardiac memory” based on the notion that the ventricular cone can be unwrapped to show a myocardial band extending from the pulmonary to the aortic root. The concept of the myocardial band was itself developed by Torrent Guasp, and has subsequently been championed by Buckberg. Neither Torrent Guasp, when formulating his initial concept, nor Buckberg in his subsequent endorsements, have validated the results of dissection using histological or other techniques that would reveal the boundaries of the alleged band. In contrast, there is a wealth of evidence showing that such boundaries do not exist and that the cardiomyocytes are packed together within the walls of the ventricular cone in the form of a three-dimensional mesh. The evidence demonstrating the manner of packing of the cardiomyocytes within the ventricular walls was summarised in another recent review published in the journal. It is disappointing that Cirillo chose to ignore the wealth of evidence disproving the concept on which he bases his entire review. Only by recognising the existence of this evidence can we truly understand ventricular function correctly, as envisaged by Cirillo.

## 1. Introduction

When summing up, in his recent review [1], the memory of the heart during its development, Cirillo states, “Some of these perspectives are purely speculative but for most of them there are already important early evidences”. The basis for the majority of his interpretations, however, is the notion of the helical heart put forward by Torrent Guasp [2]. Cirillo neglects to document the fact that, when formulating this concept, Torrent Guasp never validated his findings, obtained using dissection, by any supplementary tecnhnique that would have revealed the boundaries of the alleged myocardial band. Nor does Cirillo discuss the fact that, in the several subsequent reviews provided by Buckberg and his colleagues, as cited by Cirillo as substantiating the findings of Torrent Guasp, these authors similarly failed to provide evidence to show that the initial dissections were made by following pre-existing anatomical pathways, rather than being performed at the whim of the prosector. It is even more disturbing that Cirillo then makes no reference to the multiple investigations made by numerous observers. The studies provide compelling evidence to show that the results of the dissections of Torrent Guasp were without any anatomical foundation. 

## 2. Discussion

In his acknowledgements, Cirillo comments “We cannot fail to be grateful to all the scientists, cited and not cited, who have worked for centuries on the structure and function of the heart”. It is, however, those who have not been cited who have provided evidence, as opposed to speculation, “to understand it correctly”. It is particularly disturbing that Cirillo should conclude that Pettigrew [3] provided evidence supporting the concept as subsequently espoused by Torrent Guasp. Cirillo chooses to ignore the key sentence in the cited reference from Pettigrew: namely, that “Unlike the generality of voluntary muscles, the fibres of the ventricles, as a rule, have neither origin nor insertion, that is, they are continuous alike at the apex of the ventricles and at the base.” [3] This misinterpretation is then compounded when it is suggested that the dissection made by Pettigrew, and illustrated in Figure 2 provided by Cirillo, is comparable to the “myocardial band” as revealed by the dissections of Torrent Guasp. Torrent Guasp had unwrapped the ventricular cone to create a “band” extending from the pulmonary trunk to the aorta, as shown in the right hand panel of Figure 2 provided by Cirillo [1]. The dissection made by Pettigrew, and shown in the left hand panel of Figure 2, has the orifice of the tricuspid valve, rather than the pulmonary root, supported by the margins of the right ventricular wall. The pulmonary root can be seen in its usual position adjacent to the aortic root. The dissection of Pettigrew, therefore, is incompatible with that produced by Torrent Guasp. Pettigrew, furthermore, also produced multiple dissections showing the well-recognised helical layering of the ventricular walls. At no point in his extensive writings did he suggest that the ventricular mass could be unwrapped in the form of a continuous strand. In a recent review published in the journal directly addressing the issue of ventricular mural architecture, my colleagues and I pointed to the fact that Torrent Guasp himself had collaborated with Streeter in emphasising the helical configuration of the cardiomyocytes aggregated together within the ventricular walls [4]. We cited a key passage from the subsequent conclusion of Streeter, namely, “The heart wall was shown to be a three dimensional continuum made up essentially of the one-dimensional rod element, the cardiac muscle cell.”

As we emphasised in our review, there are multiple anatomic investigations, carried out by means of dissection, histology, and more recently by techniques such as computed tomography and diffusion tensor magnetic resonance imaging, which confirm the opinion of Streeter that the ventricular walls are arranged in the form of a three-dimensional myocardial mesh. Cirillo himself comments on the value of the newer techniques, arguing that “Thanks to the more widespread use of these two techniques, an increasing number of literature dealt with the study of cardiac layers structure and function in normal and diseased heart.” He fails to appreciate, again as emphasized in our own review, [4] that the techniques, as yet, lack the resolution to show either the individual cardiomyocytes or the fashion in which they are aggregated together. This is significant because, as was emphasized by Streeter, the working unit of the myocardium is the cardiomyocyte. The individual cardiomyocytes are then aggregated together to form units, themselves often described as “lamellae” or “sheetlets”. It is the aggregated units, forming chains, which spiral when traced throughout the ventricular walls. No investigation of which I am aware, however, has shown by means of following the aggregation of the individual cardiomyocyes that the chains can be traced so as to form the structure dissected by Torrent Guasp.

It is also disturbing that Cirillo begins his review by suggesting that cardiac looping provides the basis for the subsequent formation of the alleged band. As was shown by the recent study of Captur and colleagues, formation of the compact components of the ventricular walls is a late event during cardiac development. [5] As yet, again to the best of my knowledge, it has not been shown how, during ongoing development, the individual cardiomyocytes become aggregated together within the ventricular walls.

## 3. Conclusion

When considering ventricular mural architecture, as Cirillo summarized, we need “to understand it correctly”. This must surely be achieved when the understanding is based on evidence as opposed to speculation. Such evidence is singularly lacking from the extensive review of Cirillo. In producing his review, it is would have been difficult for him to avoid the numerous publications demonstrating, on the base of anatomic evidence, that the ventricular cone is not arranged in the fashion proposed by Torrent Guasp. In addition to the review published in this journal [4], my colleagues and I have summarised the anatomic and physiological evidence underscoring the concept of the three-dimensional mesh in two further reviews [6,7] One is compelled to ask why Cirillo chose to ignore completely the compelling evidence summarised within these reviews [4,6,7].
